# Modification of the structural and electrical properties of graphene layers by Pt adsorbates

**DOI:** 10.1088/1468-6996/15/5/055002

**Published:** 2014-09-08

**Authors:** M Waqas Iqbal, M Zahir Iqbal, M Farooq Khan, Xiaozhan Jin, Chanyong Hwang, Jonghwa Eom

**Affiliations:** 1 Department of Physics and Graphene Research Institute, Sejong University, Seoul 143-747, Korea; 2 Center for Nanometrology, Korea Research Institute of Standards and Science, Daejeon 305-340, Korea

**Keywords:** graphene, metal adsorbates, Pt doping, Raman spectroscopy, transport

## Abstract

The properties of graphene are strongly affected by metal adsorbates and clusters on graphene. Here, we study the effect of a thin layer of platinum (Pt) metal on exfoliated single, bi- and trilayer graphene and on chemical vapor deposition-grown single-layer graphene by using Raman spectroscopy and transport measurements. The Raman spectra and transport measurements show that Pt affects the structure as well as the electronic properties of graphene. The shift of peak frequencies, intensities and widths of the Raman bands were analyzed after the deposition of Pt with different thicknesses (1, 3, 5 nm) on the graphene. The shifts in the G and 2D peak positions of the Raman spectra indicate the n-type doping effect by the Pt metal. The doping effect was also confirmed by gate-voltage dependent resistivity measurements. The doping effect by the Pt metal is stable under ambient conditions, and the doping intensity increases with the increasing Pt deposition without inducing a severe degradation of the charge carrier mobility.

## 1. Introduction

In the last few years, graphene, a flat monolayer of carbon atoms arranged in a hexagonal network, has had tremendous attraction for researchers due to fascinating properties such as its very high mobility and quantum electronic transport [1–3]. However, the absence of a band gap in pristine graphene makes it unsuitable for digital device applications [4, 5]. There are still many obstacles to overcome for graphene to be adopted as a device material. Recently, the doping of graphene has drawn much interest because it is crucial to fabricate integrated devices such as logic circuits [6]. Recently, metal adatoms and clusters on graphene have been a topic of great interest since they can locally dope or modify the band structure [7, 8]. The interaction of electrons in graphene with surface adsorbates like metals and molecules is an important issue for high electronic mobility, doping and applications in sensors [9, 10]. The metal adatoms can also induce the major structural deformation of graphene. It has already been found that the absorption of metal nanoparticles changes the structural and electronic properties of graphene [11, 12]. Metals on graphene surfaces are employed as an electrical contact, which is an essential device element. Therefore, it is important to understand their influence on the structure and electronic properties of graphene. In general, a Pt contact is used as an electrode in many graphene devices. However, the physics implicated at the interface between the metallic electrodes and graphene remains ambiguous. The modification of defects in graphene by other metal adatoms has already been studied. However, no paper is available for study on the interaction of a thin layer of Pt film on graphene. Using the density functional theory, Giovannetti, Khomyakov and their co-workers extensively studied the electronic structure and charge transfer of graphene/metal interfaces. They showed that graphene is physisorbed onto Al, Ag, Cu, Au and Pt surfaces [13, 14]. However, the experimental exploration of metal/graphene systems has been limited so far, and it is important to understand their influence on the structural/electronic properties of graphene.

In this paper, we report the effect of a thin layer of Pt on exfoliated single-, bi- and trilayer graphene as well as on single-layer graphene grown by chemical vapor deposition (CVD) using Raman spectroscopy and transport measurements. The Raman spectra and transport measurements show that Pt affects the structure as well as the electronic properties of graphene. The Raman bands were analyzed before/after the deposition of Pt with different thicknesses (1, 3, 5 nm) on graphene. The Raman spectra and transport measurements indicate the n-type doping effect by Pt deposition.

## 2. Experimental section

Pt metals with different thicknesses (1, 3, and 5 nm) were deposited on exfoliated single, bi- and trilayer graphene and on single-layer CVD-grown graphene by a thermal evaporation system in a high vacuum (∼8 × 10^−7^ torr) at a rate of 1.0 Å s^−1^ The Pt deposition was monitored by a quartz oscillator and confirmed with atomic force microscopy (AFM). To avoid sample heating during deposition, the sample stage was cooled by a continuous water flow. The Raman spectra were measured before and after the metal deposition on the same graphene sample. The Raman spectra were recorded at room temperature with a Renishaw microspectrometer. The laser wavelength was 514 nm; to avoid a laser-induced heating effect, the power was kept at 1.0 mW. The diameter of the laser spot was less than 1 *μ*m. The Dirac points (or the change in the neutrality point) of the graphene samples were observed by a gate-voltage dependent resistivity measurement using a 4-probe configuration method with a lock-in amplifier at room temperature.

The exfoliated single, bi- and trilayer graphene were obtained from the bulk graphite using a standard Scotch Tape method and were confirmed with optical and Raman spectra, whereas the CVD-grown single-layer graphene was obtained using thermal CVD. The growth process of the graphene is as follows: The graphene used in this study was grown on 25 *μ*m thick copper foils from Alfa Aesar (99.8% pure) via thermal CVD. A mechanically polished and electropolished copper foil was inserted into a thermal CVD furnace. The furnace was evacuated to ∼10^–4^ torr and heated to 1010 °C under an H_2_ gas flow (∼10^–2^ torr). After the temperature became stable at 1010 °C, both the CH_4_ (20 standard cubic centimeters per minute (sccm)) and the H_2_ (5 sccm) were injected into the furnace for 8 min to synthesize the graphene. After the graphene synthesis, the sample was cooled at a rate of 50 °C min^−1^ to room temperature [15, 16]. The grown graphene film was then transferred to the SiO_2_/Si wafer as follows: The Cu foil was etched in an aqueous solution of ammonium persulfate (APS). The surface of the graphene was spin-coated with polymethyl methacrylate (PMMA), and the sample was then baked at 70 °C for 10 min The PMMA coating was applied to prevent the graphene films from cracking and folding during the transfer to a desired substrate. The PMMA/graphene film was washed with deionized water after the Cu foil had been completely dissolved; it was then transferred onto the Si/SiO_2_ wafer. The PMMA film was removed with acetone. The graphene sample was subsequently cleaned in isopropanol and dried in a N_2_ flow [15, 16].

## 3. Results and discussion

**Figure 1.**
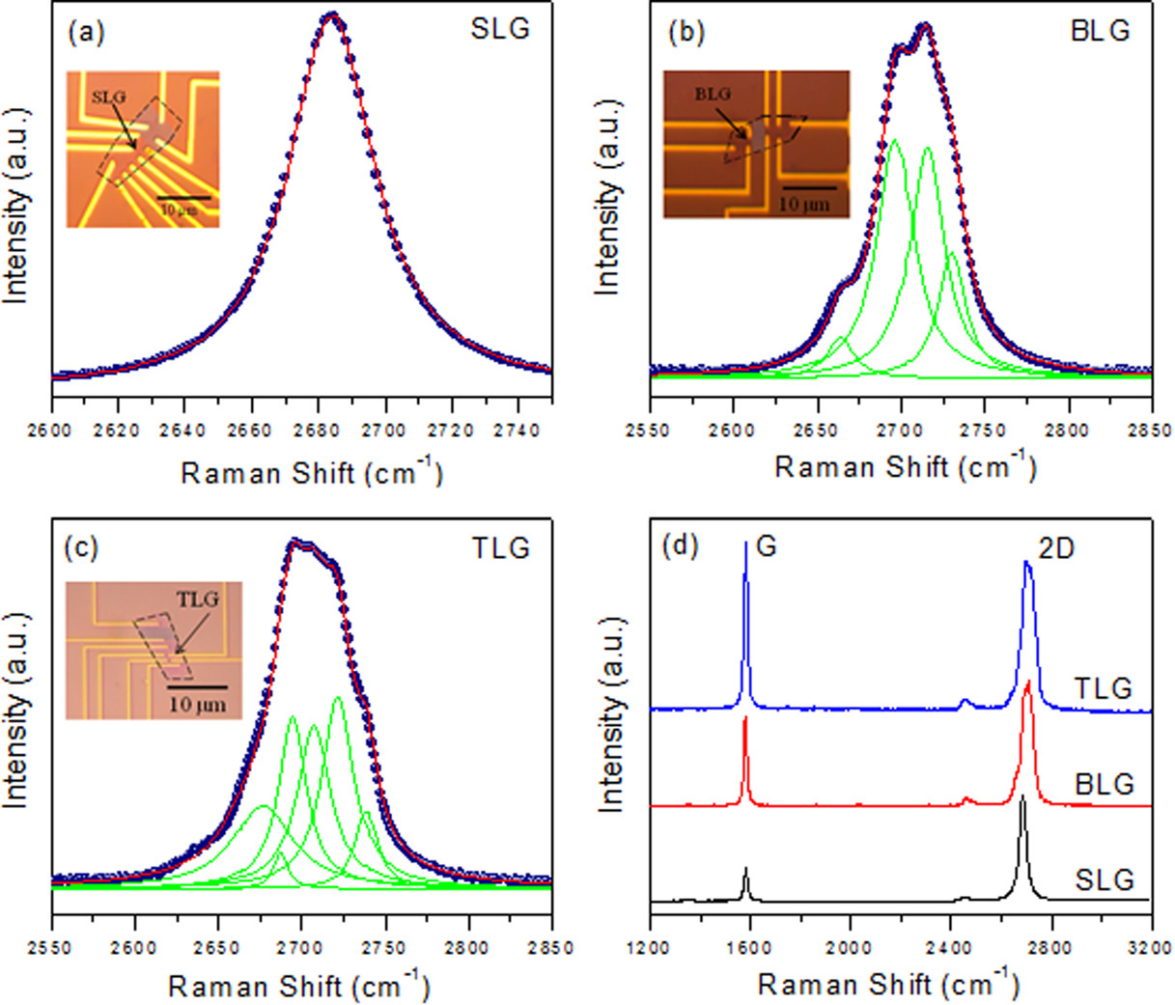
(a) Single Lorentzian fit of the 2D peak for the SLG. (b) A four Lorentzian curve fitting of the broad 2D peak of the BLG. (c) A six Lorentzian curve fitting of the broad 2D peak of the TLG. (d) Raman spectra of the pristine exfoliated SLG, BLG and TLG samples.

**Figure 2.**
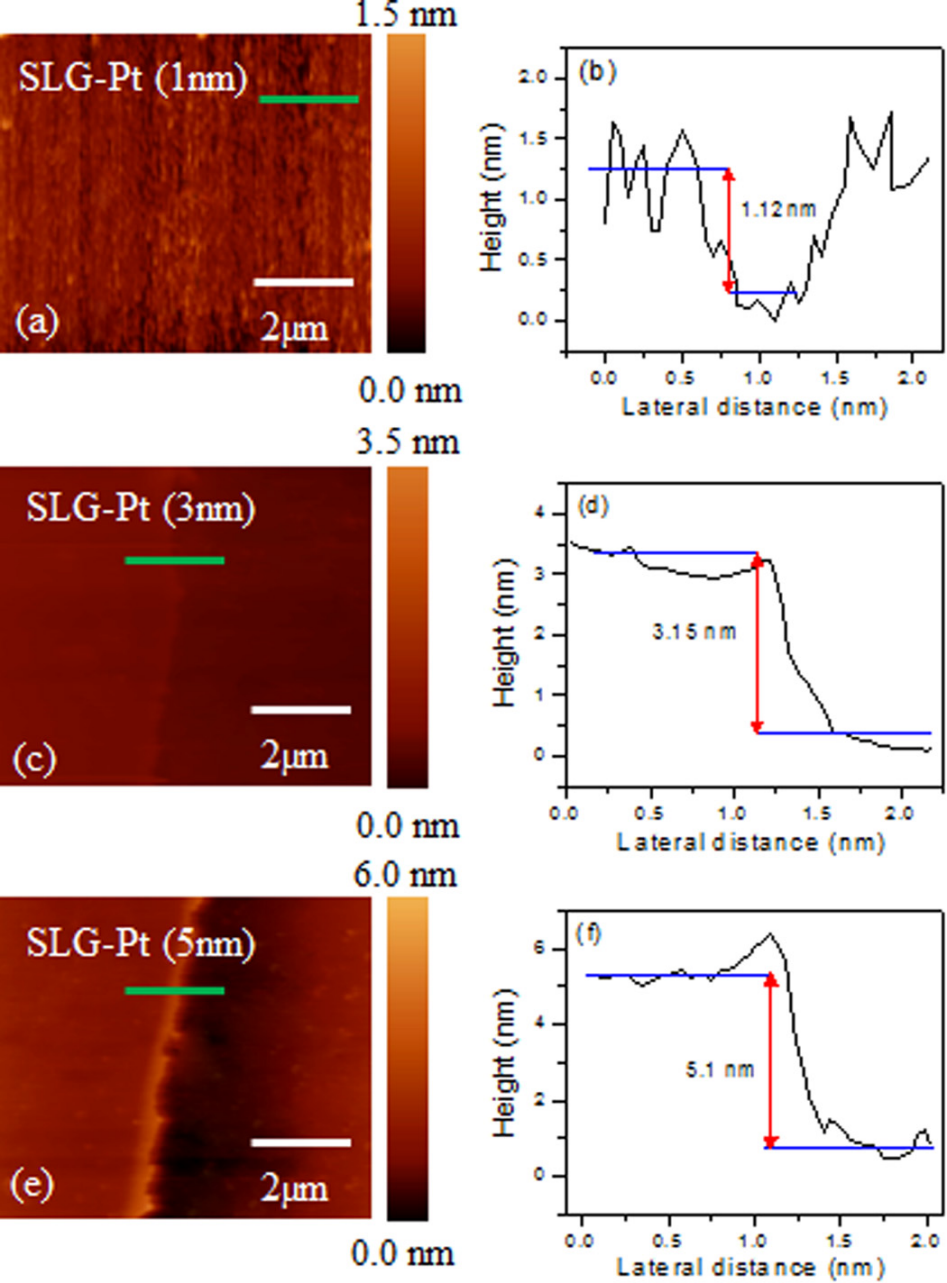
(a) AFM image of CVD-grown graphene after 1 nm Pt deposition. (b) AFM image line profile of CVD-grown graphene after 1 nm Pt deposition. (c) AFM image of CVD-grown graphene after 3 nm Pt deposition. (d) AFM image line profile of CVD-grown graphene after 3 nm Pt deposition. (e) AFM image of CVD-grown graphene after 5 nm Pt deposition. (f) AFM image line profile of CVD-grown graphene after 5 nm Pt deposition.

Figure 1 shows the Raman spectra of the exfoliated pristine single-layer graphene (SLG), the bilayer graphene (BLG) and the trilayer graphene (TLG). Figure 1(a) shows the single Lorentzian fit of the 2D peak for the SLG. A broad 2D peak is fitted with four Lorentzian curves, as can be seen in figure 1(b), which confirms the BLG. Figure 1(c) shows the fitting of the broad 2D peak in the TLG by six Lorentzian curves. Figure 1(d) shows the Raman spectra of the exfoliated pristine SLG, BLG and TLG sample. The absence of the D peak in all of the samples indicates the high quality of our samples. We used AFM to measure the thickness and morphology of each graphene sample after Pt deposition. Figure 2 depicts the surface topology and line profile of the graphene by using AFM after 1, 3 and 5 nm Pt deposition on the CVD-grown graphene. All scans were taken in a tapping mode under ambient conditions, and the scan area was kept at 10 × 10 *μ*m. The line profiles of the graphene sample after 1, 3 and 5 nm Pt deposition are shown in figure 2. The line profiles were obtained across the boundary between the deposition area and covered area. However, the boundary is not clear for the 1 nm deposition in figure 2(a). The thickness measurement by the AFM is consistent with the nominal thickness measured by the quartz oscillator (thickness monitor) for the Pt film deposition. The thicknesses of the graphene samples after 1, 3 and 5 nm Pt deposition were measured as 1.1, 3.2 and 5.1 nm, as seen in figures 2(b), (d) and (f).

**Figure 3.**
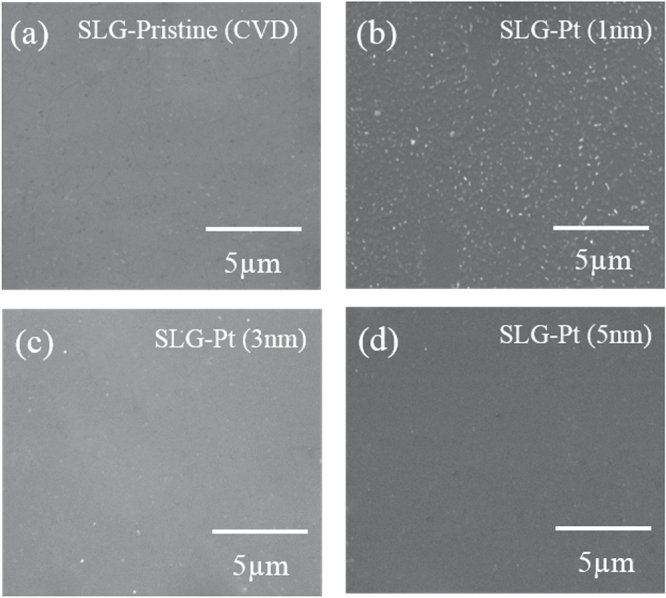
(a) SEM image of pristine CVD-grown graphene. (b) SEM image of CVD-grown graphene after 1 nm Pt deposition. (c) SEM image of CVD-grown graphene after 3 nm Pt deposition. (d) SEM image of CVD-grown graphene after 5 nm Pt deposition.

The Pt film’s morphology is examined in figures 2(a), (c) and (e). The deposition is not uniform and shows clusters for the 1 nm Pt deposition, as shown in figure 2(a), whereas the films become more uniform and smooth for the 3 and 5 nm Pt deposition. The morphology of the Pt films is also examined using scanning electron microscopy (SEM). Figure 3(a) shows the SEM image of the pristine CVD-grown graphene. The SEM image clearly shows the high quality of the pristine graphene with little residue. The SEM image of the CVD-grown graphene after the 1 nm Pt deposition shows clusters all over the graphene’s surface, as shown in figure 3(b). For the 3 and 5 nm Pt deposition, the films are uniform and continuous, as seen in figures 3(c) and (d).

**Figure 4.**
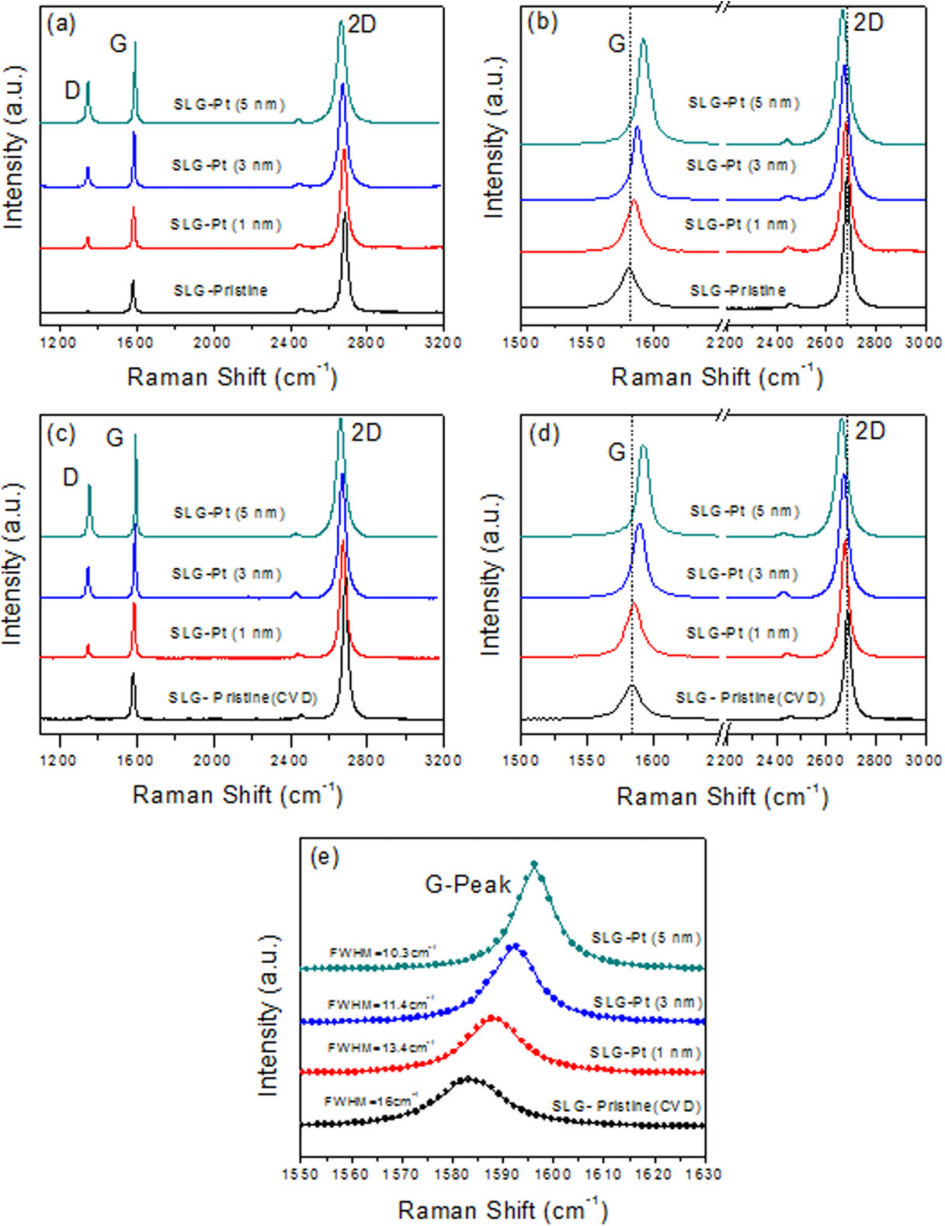
(a) Raman spectra of pristine exfoliated SLG before and after 1, 3 and 5 nm Pt deposition. (b) Detailed Raman spectra for the G and 2D of pristine exfoliated SLG before and after 1, 3 and 5 nm Pt deposition. (c) Raman spectra of pristine CVD-grown SLG before and after 1, 3 and 5 nm Pt deposition. (d) Detailed Raman spectra for the G and 2D of pristine CVD-grown SLG before and after 1, 3 and 5 nm Pt deposition. (e) Lorentzian fit of the G peak for CVD-grown SLG (pristine graphene) before and after 1, 3 and 5 nm Pt-coated samples. The circles represent the experimental data, while the lines represent the Lorentzian fit for the G peak. The FWHM was ∼16 cm^−1^ for the pristine graphene and 13.4, 11.4 and 10.3 cm^−1^ for the 1, 3 and 5 nm Pt-coated graphene samples, respectively.

The Raman spectra of the exfoliated and CVD-grown SLG before and after the 1, 3 and 5 nm Pt deposition are shown in figure 4. The G and 2D peaks of the pristine exfoliated SLG are observed at 1581 cm^−1^ and 2683 cm^−1^, respectively, as shown in figures 4(a) and (b). These figures also show the Raman spectra of the exfoliated SLG after the 1, 3 and 5 nm Pt deposition. A change in the intensities and the full width at half maximum (FWHM) of the G, 2D and D peaks are observed after the 1, 3 and 5 nm Pt coating. The *I*_*D*_/*I*_*G*_ ratio increases in the exfoliated SLG from 0.01 to 0.3 after the deposition of 1 nm of Pt. The ratio of *I*_2*D*_/*I*_*G*_ becomes 2.39 compared to 3.30 in pristine SLG. After the 3 nm Pt deposition on the exfoliated SLG, the ratio of the *I*_2*D*_/*I*_*G*_ is significantly reduced to 1.86. The ratio of the *I*_*D*_/*I*_*G*_ is 0.41 for the exfoliated SLG after 3 nm of Pt metal deposition. The 2D band is a single peak at 2671 cm^−1^, which is red- (downward) shifted by 13 cm^−1^ from the pristine graphene. On the other hand, the G band of the graphene after the Pt deposition is found to be at 1587 cm^−1^, which is blue- (upward) shifted by 6 cm^−1^. After the 5 nm Pt metal deposition, a more pronounced D band peak is observed. The ratio of *I*_*D*_/*I*_*G*_ is 0.50 for the graphene after 5 nm of Pt deposition. The intensity of the 2D band is significantly reduced, and the ratio of the *I*_2*D*_/*I*_*G*_ becomes 1.25. The 2D band is observed at 2665 cm^−1^, which is red-shifted by 19 cm^−1^ from the pristine graphene. The FWHM of the G peak has been observed at 15.5, 13.1, 10.2 and 9.7 cm^−1^ before and after the 1, 3 and 5 nm Pt metal deposition, respectively. The blue shift of the G peak, the red shift in the 2D peak and the reduction of the FWHM of the G peak indicate an n-doping effect [17–21], which is also confirmed by the electrical measurement of the graphene device. For further verification, we also analyzed the CVD-grown SLG by using the same metal of the 1, 3 and 5 nm thick Pt film. Figures 4(c) and (d) show the Raman spectra of the CVD-grown SLG before and after the 1, 3 and 5 nm Pt deposition. The G band was observed at 1582 cm^−1^ for the pristine CVD-grown SLG, whereas it was found at 1585, 1588 and 1591 cm^−1^ for the 1, 3 and 5 nm Pt deposition, respectively. The ratio of *I*_*D*_/*I*_*G*_ was changed to 0.28, 0.37 and 0.47 after the 1, 3 and 5 nm Pt deposition, respectively, as compared to the 0.02 ratio for the pristine CVD-grown graphene. The 2D band of the pristine graphene was observed at 2684 cm^−1^, whereas those after the Pt metals with thicknesses of 1, 3 and 5 nm were observed at 2679, 2674 and 2668 cm^−1^, respectively, which is red-shifted by 16 cm^−1^ from the pristine CVD-grown graphene. The ratio of *I*_2*D*_/*I*_*G*_ was changed to 2.3, 1.9 and 1.35 after the 1, 3 and 5 nm Pt deposition, respectively, compared to the 2.9 ratio for the pristine CVD-grown graphene.

Both the n- and p-type doping can lead to the reduction of the FWHM of the G-peak, as already reported in [18]. We have done a Lorentzian fitting of the G-peak for the pristine 1, 3 and 5 nm Pt-coated CVD-grown graphene samples, as shown in figure 4(e). The FWHM of the G peak has been observed as 16, 13.4, 11.4 and 10.3 cm^−1^ before and after the 1, 3 and 5 nm Pt metal deposition, respectively. We found that the FWHM of the G-peak decreased after the Pt deposition. However, the strain usually results in an increase of the FWHM of the G-peak, as reported in [22]. Furthermore, it was reported that the G-peak splits into two distinct G and G’ peaks if the strain is large enough. It was also reported that the increasing strain resulted in the red shift for both the G and 2D peak positions [22]. However, in this experiment, we observed a blue shift in the G peak position, a red shift in the 2D peak position and a reduction of the FWHM of the G peak position after the Pt deposition. Therefore, we conclude that the shift of the Raman peaks in this experiment is due to doping rather than strain [17–21]. We note that the exfoliated graphene is more subject to n-type doping than is the CVD-grown graphene. We believe some of the organic residues or impurities that formed during the transfer process hindered the coupling between the Pt and carbon atoms of the graphene.

**Figure 5.**
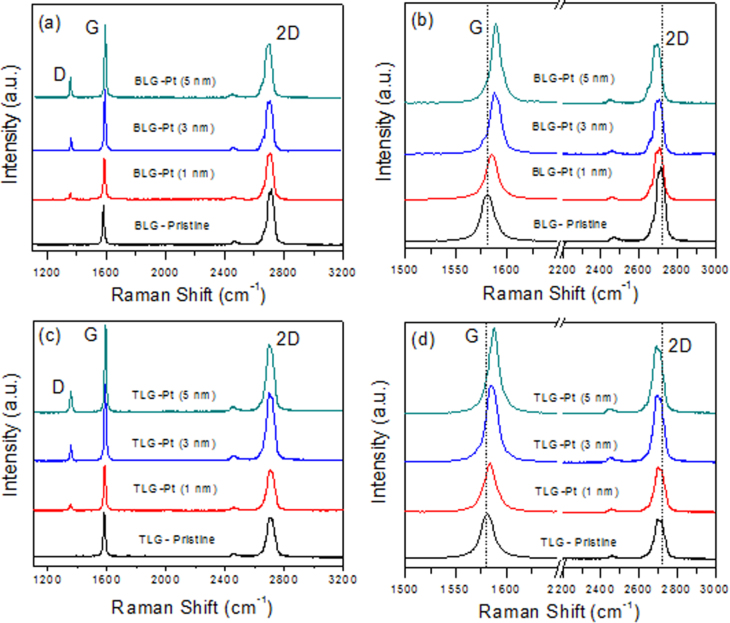
(a) Raman spectra of the pristine exfoliated BLG before and after the 1, 3 and 5 nm Pt deposition. (b) Detailed Raman spectra for the G and 2D of pristine exfoliated BLG before and after the 1, 3 and 5 nm Pt deposition. (c) Raman spectra of the pristine TLG before and after the 1, 3 and 5 nm Pt deposition. (d) Detailed Raman spectra for the G and 2D of pristine TLG before and after the 1, 3 and 5 nm Pt deposition.

Figures 5(a) and (b) show the Raman spectra of the exfoliated BLG before and after the 1, 3 and 5 nm Pt deposition. Before the Pt deposition, the G band was observed around 1581 cm^−1^ for the exfoliated BLG. On the other hand, the G band of the bilayer graphene after the 1, 3 and 5 nm Pt deposition was found to be at 1583 cm^−1^, 1586 cm^−1^ and 1589 cm^−1^. The ratio of I_2D_/I_G_ became 0.90 after the 5 nm Pt deposition, whereas it was 1.36 in the pristine BLG. The 2D peak was found at 2705 cm^−1^, 2701 cm^−1^ and 2696 cm^−1^ after the 1, 3 and 5 nm Pt deposition, respectively. The red shift of 11 cm^−1^ in the 2D band was found in the exfoliated BLG after the 5 nm Pt deposition. It is known that the blue shift of the G band positions and the red shift of the 2D band positions indicate the n-doping of the graphene. Figures 5(c) and (d) show the Raman spectra of the exfoliated TLG before and after the 1, 3 and 5 nm Pt deposition. Before the Pt deposition, the G band was observed to be around 1582 cm^−1^ for the exfoliated TLG. On the other hand, the G band of the graphene after the 1, 3 and 5 nm Pt deposition was at 1584, 1585.5 and 1587 cm^−1^. The ratio of *I*_2*D*_/*I*_*G*_ became 0.65 compared to 0.8 in pristine TLG. The red-shifted 2D peaks were found at 2706 cm^−1^, 2703 cm^−1^ and 2699 cm^−1^ after the 1, 3 and 5 nm Pt deposition. Again, the blue shift of the G band positions and the red shift of the 2D band positions are indicative of n-doping of the TLG [4, 7, 17–21]. The n-type doping effect is also confirmed by the electrical measurement of the same graphene sample after the 1, 3 and 5 nm Pt deposition.

**Figure 6.**
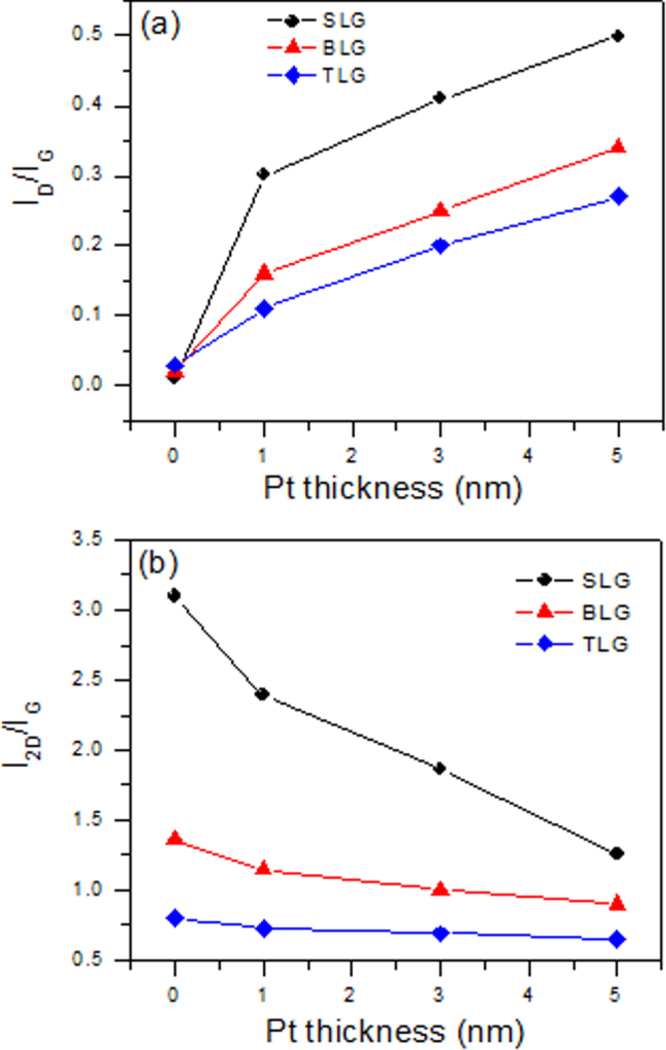
(a) *I*_*D*_/*I*_*G*_ ratio in the Raman spectra of pristine exfoliated SLG, BLG and TLG before and after the 1, 3 and 5 nm Pt deposition. (b) *I*_2*D*_/*I*_*G*_ ratio in the Raman spectra of the pristine exfoliated SLG, BLG and TLG before and after the 1, 3 and 5 nm Pt deposition.

Figure 6(a) shows the *I*_*D*_/*I*_*G*_ ratio before and after the 1, 3 and 5 nm Pt deposition in exfoliated SLG, BLG and TLG. The *I*_*D*_/*I*_*G*_ ratio for the pristine state in SLG, BLG and TLG is around zero, indicating the high quality of the graphene samples, but after the Pt deposition, the *I*_*D*_/*I*_*G*_ ratio increases significantly. A larger change is observed for the SLG compared to the BLG and TLG. The *I*_*D*_/*I*_*G*_ ratio is 0.5 in the SLG, while it is 0.25 in the TLG. Figure 6(b) shows the *I*_2*D*_/*I*_*G*_ ratio before and after the 1, 3 and 5 nm Pt metal deposition in exfoliated SLG, BLG and TLG. The largest change in the *I*_2*D*_/*I*_*G*_ ratio is observed in the SLG. The *I*_2*D*_/*I*_*G*_ ratio changes from 3.1 to 1.1 in the SLG, while the *I*_2*D*_/*I*_*G*_ ratio changes from 0.8 to 0.65 in the TLG.

**Figure 7.**
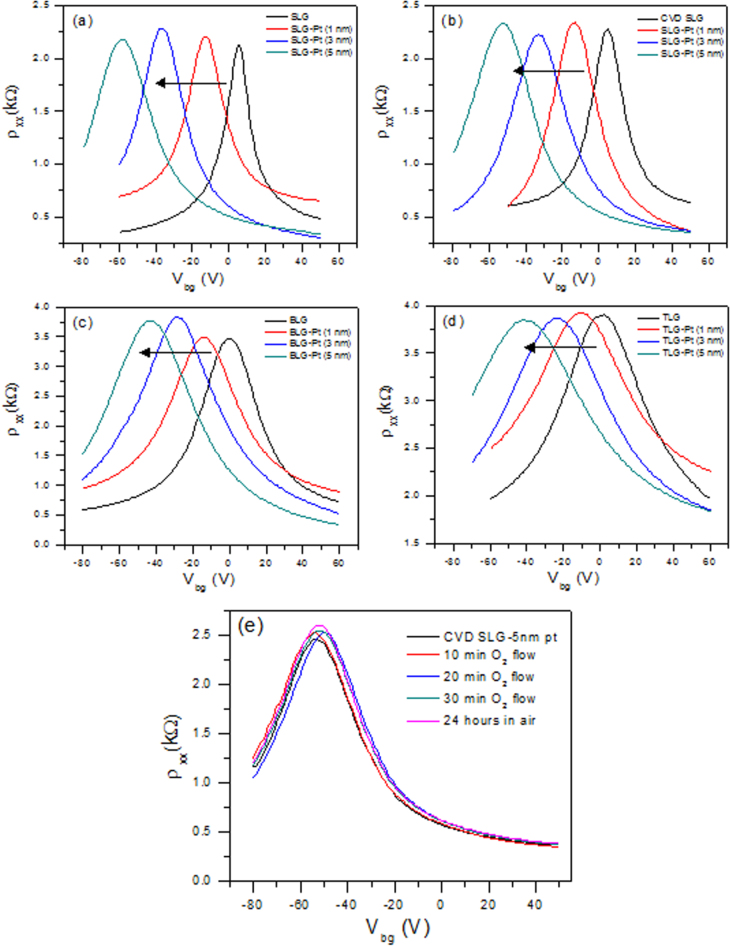
(a) Gate-voltage dependent resistivity of the pristine exfoliated SLG before and after the 1, 3 and 5 nm Pt deposition. (b) Gate-voltage dependent resistivity of the pristine CVD-grown SLG before and after the 1, 3 and 5 nm Pt deposition. (c) Gate-voltage dependent resistivity of the pristine exfoliated BLG before and after the 1, 3 and 5 nm Pt deposition. (d) Gate-voltage dependent resistivity of the pristine exfoliated TLG before and after the 1, 3 and 5 nm Pt deposition. (e) Dirac point of CVD single-layer graphene with a 5 nm Pt deposition after exposure to oxygen and air for different time periods.

Figure 7 shows the gate-dependent resistivity of the pristine exfoliated SLG, BLG, TLG and CVD-grown single-layer graphene. The maximum in the gate-dependent resistivity identifies the back gate-voltage (V_g_), which corresponds to the Dirac point, while the slope indicates the mobility of the charge carriers in the graphene [23–25]. The Dirac point of the pristine graphene sample is found around *V*_*g*_ = 0 V, indicating an undoped feature of the pristine graphene. The deposition of Pt on the graphene surface causes the shift of the Dirac point toward negative gate-voltages, showing the *n*-type doping effect [26, 27]. The Dirac points were shifted from 5 V to −12, −36 and −57 V after the 1, 3 and 5 nm Pt deposition in the exfoliated SLG, as shown in figure 7(a). The Dirac points shifted toward more negative gate-voltages as we increased the Pt thicknesses, which increased the n-type doping. For further verification, we also measured the Dirac points for the CVD-grown SLG. In figure 7(b), the Dirac points were observed at −13, −32 and −52 V after the 1, 3 and 5 nm Pt deposition, respectively. Figure 7(c) shows the gate-voltage dependent resistivity for the pristine exfoliated BLG and the 1, 3 and 5 nm thick Pt deposited in the BLG. The Dirac points were shifted from 0 V to −15, −29 and −46 V after the 1, 3 and 5 nm Pt deposition, respectively. Figure 7(d) shows the gate-voltage dependent resistivity for the pristine exfoliated TLG and the 1, 3 and 5 nm thick Pt deposited in the TLG. The Dirac points moved from 0 V to −11, −24 and −41 V after the 1, 3 and 5 nm Pt deposition, respectively. To check the stability of the Pt doping, we also measured the sample after exposing it to an oxygen gas flow for a certain amount of time, as shown in figure 7(e). The CVD-grown SLG with the 5 nm thick Pt deposition was used for a stability check. No significant change was observed in the gate-voltage-dependent resistivity after 30 min of exposure. We also checked the stability of the Pt doping. After leaving the samples under ambient conditions for one day, their transport properties remained the same.

**Figure 8.**
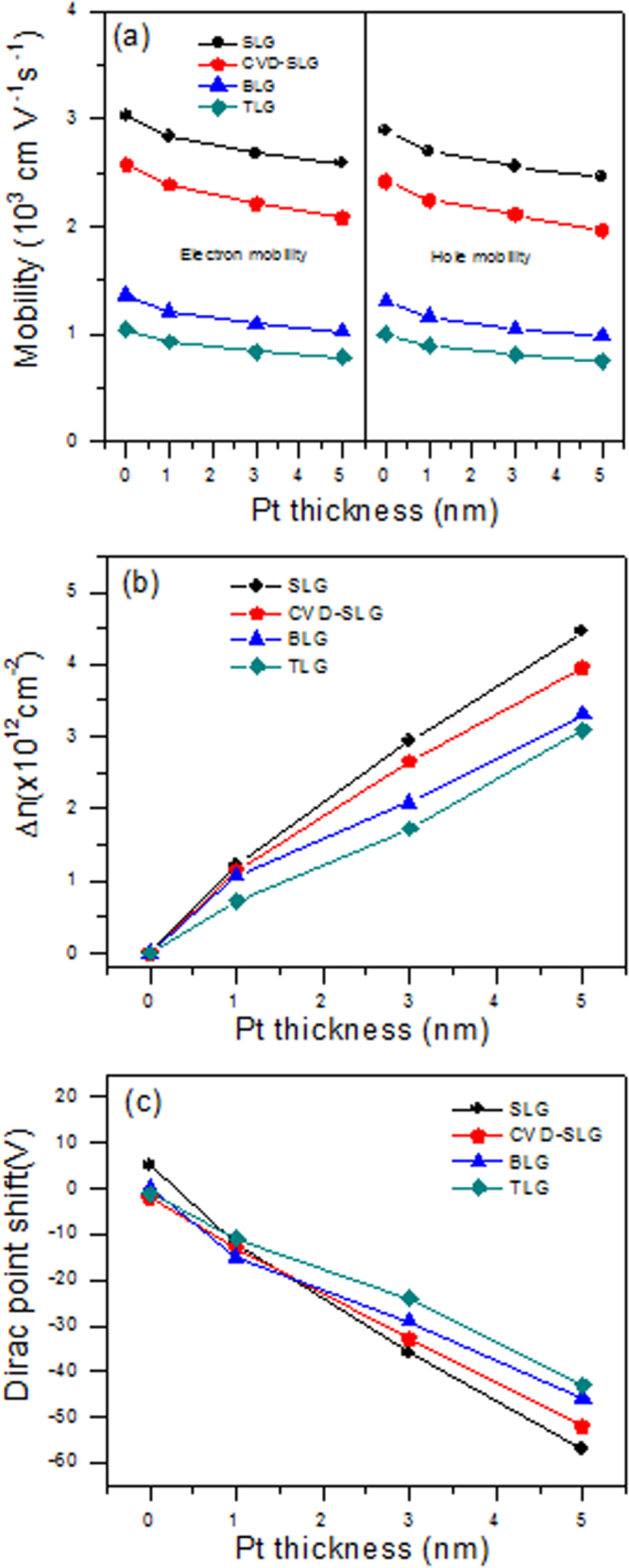
(a) The electron and hole motilities before and after the deposition of Pt metal with different thicknesses in the SLG, BLG and TLG. (b) The change in carrier concentration after the 1, 3 and 5 nm Pt deposition. (c) Change in the Dirac points for the CVD-grown SLG and the exfoliated SLG, BLG and TLG after the 1, 3 and 5 nm Pt deposition.

Figure 8(a) shows the electron and hole mobility before and after the deposition of different Pt film thicknesses. The mobility was obtained using relation *μ* = (1/C_g_)(∂*σ*/∂V_g_), where *σ* is the conductivity of the samples, and *V*_*g*_ is the gate-voltage [28–31]. The mobility of the graphene decreased slightly with the increasing Pt thickness. However, the change in the mobility of the BLG and TLG was smaller than that of the SLG with the Pt deposition, as shown in figure 8(a). Figure 8(b) shows the change in the carrier concentration after the 1, 3 and 5 nm Pt deposition. The carrier concentration increases with the increasing Pt deposition. The trend of the carrier concentration change is similar, but the largest increase was observed in the SLG. The reduced doping effect in the BLG and TLG as compared to the SLG may be due to the influence of Pt, which is dominant in the top layer of the BLG and TLG.

## 4. Conclusion

Using Raman spectroscopy and transport measurements, we studied the interaction of thin layers of Pt with exfoliated single, bi- and trilayer graphene and CVD-grown single-layer graphene. The Raman spectra and transport measurements indicate that Pt affects the structural as well as the electronic properties of the graphene. The shifts in the G and 2D peak positions indicate the n-doping of graphene by the Pt metal. The doping effect is also confirmed by gate-voltage dependent resistivity measurements. However, it was found that Pt affects the characteristics of the SLG more than the BLG or TLG. We have also verified the stability of our graphene devices under ambient conditions and in an oxygen flow.
